# Advancements in the conservation of the conformational epitope of membrane protein immunogens

**DOI:** 10.3389/fimmu.2025.1538871

**Published:** 2025-02-28

**Authors:** Aisha Mahboob, Nishat Fatma, Ahmed Faraz, Muntaha Pervez, Mohammad Afeef Khan, Afzal Husain

**Affiliations:** Department of Biochemistry, Faculty of Life Sciences, Aligarh Muslim University, Aligarh, India

**Keywords:** immunogen, membrane protein, antibody, lipid bilayer, native structure

## Abstract

Generating antibodies targeting native membrane proteins presents various challenges because these proteins are often embedded in the lipid bilayer, possess various extracellular and intracellular domains, and undergo post-translational modifications. These properties of MPs make it challenging to preserve their stable native conformations for immunization or antibody generation outside of the membranes. In addition, MPs are often hydrophobic due to their membrane-spanning regions, making them difficult to solubilize and purify in their native form. Therefore, employing purified MPs for immunogen preparation may result in denaturation or the loss of native structure, rendering them inadequate for producing antibodies recognizing native conformations. Despite these obstacles, various new approaches have emerged to address these problems. We outline recent advancements in designing and preparing immunogens to produce antibodies targeting MPs. Strategies outlined here are relevant for producing antibodies for research, diagnostics, and therapies and designing immunogens for vaccination purposes.

## 1 Introduction

The immune response is the defense mechanism of our body against substances it perceives as foreign or dangerous, generally marked as antigens (1). When the immune system detects an antigen, it attempts to attack and destroy the target molecule. The recognition molecules utilized by the immune system are either membrane-bound receptors or soluble proteins. This response has two key components, namely cellular immunity and humoral immunity. The former involves immune cells directly targeting and destroying non-self molecules, cancer cells, and whole pathogens, whereas the latter relies on B cells produced antibodies that bind to antigens, neutralizing them (2). B cells recognize solvent-exposed regions of an antigen, also called B cell epitope, that interact with both secreted and membrane-bound immunoglobulins (3). Based on their immunogenic potential B cell epitopes can be classified as immunodominant, immunogenic, and non-immunogenic (4). On the other hand, T cell epitopes are peptides derived from antigens, presented on the surface of antigen-presenting cells bound to MHC molecules, where the T cell receptor recognizes them. Most T cell epitopes are linear, whereas B cell epitopes can be either linear (10%) or conformational (90%). Therefore, preserving conformational epitopes while designing immunogens for raising antibodies is extremely important. Epitope identification in antigens is crucial for understanding disease mechanisms, immune monitoring, and designing epitope-based immunogens for both vaccines and developing antibodies for therapeutic, diagnostic, and research purposes (5). While several experimental approaches can identify B cell epitopes, including determining the three-dimensional (3D) structure of antigen-antibody complexes and screening peptide libraries for antibody binding, recently, B cell epitope prediction tools have also been developed (6).

**Figure 1:**
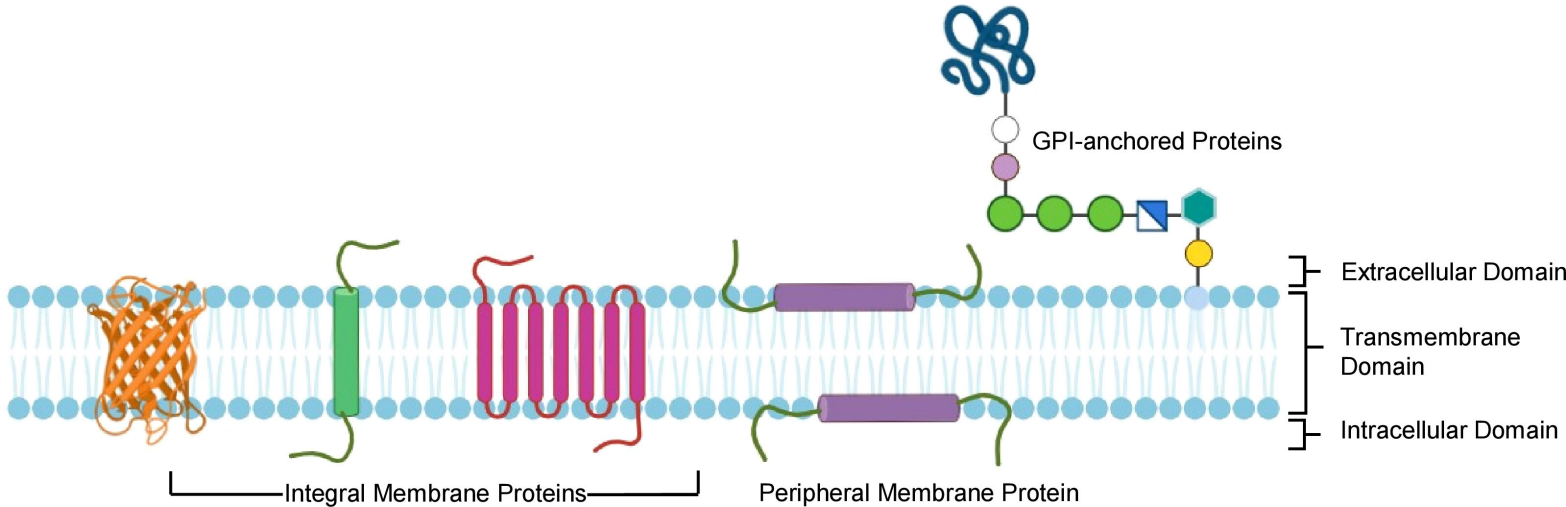
Different types of membrane proteins and their domains. Showing membrane protein structure and orientation within the lipid bilayer.

The ability of the immune system to produce specific and high-affinity antibodies against foreign antigens is harnessed for several purposes, including research and diagnostics, enabling the development of tools for detecting and analyzing biomolecules. This ability of immune system is also used in vaccination, where long-term disease prevention is achieved by stimulating the creation of memory cells and antibodies in response to exposure to a harmless form of an antigen (7, 8). Antibodies are indispensable for advancing our understanding of membrane protein's (MPs) structure, function, localization, transport, and interaction with various ligands (9). MPs account for over 20-30% of all cellular proteins encoded by the human genome and are essential for numerous cellular functions, including material transport, signal transduction, intercellular recognition, ligand-receptor binding, and cell adhesion, making them crucial therapeutic targets (10). MP can exist in various forms characterized by hydrophobic transmembrane spanning domain and including cell signaling receptors such as G protein-coupled receptors (GPCRs), ion channels, transporters, tight junction proteins, and signaling molecules, however, they remain underrepresented in Protein Data Bank, a worldwide repository for structural data (**Figure 1**) (9, 11–13). Advances in protein production and the secretion capacities of microbial hosts have positioned MPs at the forefront of therapeutic research, with MPs accounting for over 60% of current drug targets for various ailments (14). With the rapidly evolving nature of pathogens, as seen by SARS-CoV-2 during the pandemic, the efficient soluble production and study of MP domains involved in pathogenicity have become critical. The variations in the MPs that are target of the immune system against pathogens often possess characteristics that enable them to evade immune responses and antibody treatments, posing significant threats to public health. Since MP serves as a key entry point for critical molecules and pathogens, antibody production against MP immunogen has therapeutic advantages (15). MPs role in cellular interactions and pathogen recognition, when used as immunogens, can specifically elicit immune responses against pathogens. As a result, they hold significant potential for use in vaccine development and in generating antibodies for diagnostics and therapeutic applications. Despite numerous applications of antibodies in understanding MP biology, the structural intricacy of MPs makes it difficult to produce high-quality antibodies against them. It is challenging for MPs to retain their native conformation outside the membrane or their natural environment due to their hydrophobic regions that are naturally embedded in the lipid bilayer (16). Moreover, MPs only reveal a small fraction of their structure on the cell surface, which limits the accessibility of the epitopes available to generate antibodies (17). These restrictions make it more challenging to describe MPs and create efficient treatments targeting them thoroughly.

Traditional methods for generating MP antibodies, such as immunization with reconstituted MP or peptides, face several drawbacks, such as limited 3D presentation of MPs, their instability in purified forms, and the restricted solvent exposure of accessible epitopes. Conformational epitopes, essential for functional antibodies, are frequently missed in peptide-based immunization, making these antibodies unsuitable for applications like MP co-crystallization (18). Exposure of MPs to non-native environments can induce conformational changes, leading to low-resolution structures and potential misinterpretation of their structure and function when isolated as pure proteins. Purification of MP such as GPCR, N-methyl-D-aspartate (NMDA) receptors, and epidermal growth factor receptor (EGFR) frequently disrupts the lipid-protein interactions, membrane anchoring, and dimerization necessary to maintain their active conformations. As a result, these proteins lose their structural integrity, making them unsuitable for immunization (19, 20). For instance, *Methanocaldococcusjannaschii* S2P (MjS2P) protein crystallized in detergent showed multiple conformations, complicating the identification of its physiological structure (21). Similarly, γ-secretase reconstituted in an amphipol A8-35, a class of amphiphilic polymers that make it possible to keep MP soluble in detergent-free aqueous solution, yielded a high-resolution structure, whereas detergent use resulted in lower resolution (22). Another bottleneck for generating antibodies against MPs is the lack of effective screening methods that utilize MPs in their native environment.

Recent advancements in technology have improved the strategies for immunogen designing and preparation while enhancing the efficiency of antibody production and screening. Development of new strategies relying on membrane-based and nanoparticle-based technologies, genetic immunization, and flow cytometry-based transfection have shown promising results in MP immunization and antibody development. In addition, advancements in computational methods have significantly enhanced immunogen design by enabling the analysis of protein sequences, structural modeling, and the assessment of immunogenicity and antigenicity. Artificial Intelligence (AI) based structural prediction tools, such as AlphaFold2, have shown great potential to improve immunogen design processes by providing more accurate structural prediction of MPs (23).

This review outlines recent advancements in strategies for immunogen design for developing antibodies against MPs. Full-length MPs or their specific extra-cellular domains (ECDs) can be purified in their native form or reconstituted into desired membrane mimetics for use as immunogens. The commonly used membrane mimetics are categorized into two approaches: detergent- and nanoformulation-based strategies, which use purified proteins; and membrane-based strategies, which express the target Protein in its native lipid environment and avoid protein purification. The immunogen design and preparation strategies discussed here are crucial for producing antibodies for research, diagnostics, therapeutic applications, and designing immunogens for vaccination purposes.

## 2 Stabilizing the conformation of purified proteins

Soluble proteins have long been used as antigens to immunize animals to generate antibodies as they are easy to produce and stimulate both humoral and cell-mediated immunity. Many factors influence the use of soluble proteins as immunogens; the most crucial is their inability to retain their native conformation and function during the purification and preparation of immunogens, which is essential to produce antibodies that recognize the native proteins (24). The successful production of antibodies using any method will depend on the accessibility of epitopes on the target MP. However, using full MP as an immunogen is considered more suitable for producing antibodies than focusing on the ECD because full MP may present unique conformational epitopes that are not accessible or lose their native structure in truncated extracellular fragments. Moreover, ECD also result in low immunogenicity because of their conserved sequence (25). In most cases, MPs are purified using recombinant methods and extracted using detergents to solubilize the proteins in detergent micelles (26). To keep MP in its native environment, purified proteins are incorporated into liposomes to form proteoliposomes. Although detergents, micelles, and liposome-based methods are extremely helpful for solubilization and stabilization of MPs for immunogen generation, these approaches come with their own notable limitations. For instance, detergents and micelles often ablate critical protein-lipid interactions due to their inability to mimic the native lipid environment. Similarly, despite the closeness of liposome-based methods in mimicking natural lipid bilayers, they present challenges due to the aggregation tendency of the membrane, making it difficult to obtain proteoliposomes in a homogenous and stable state (27). Another approach to assemble and stabilize the membrane structure involves incorporating detergent-solubilized and purified MPs into nanoparticles such as nanodiscs, Saposin lipid nanoparticles (SapNPs), and Styrene-maleic acid-lipidparticles (SMALPs) into an artificial bilayer mimicking the native environment (28). In addition, nano-based platforms also facilitate antibody discovery, validation, and characterization through ELISA and surface plasmon resonance.

### 2.1 Detergent micelles

**Figure 2:**
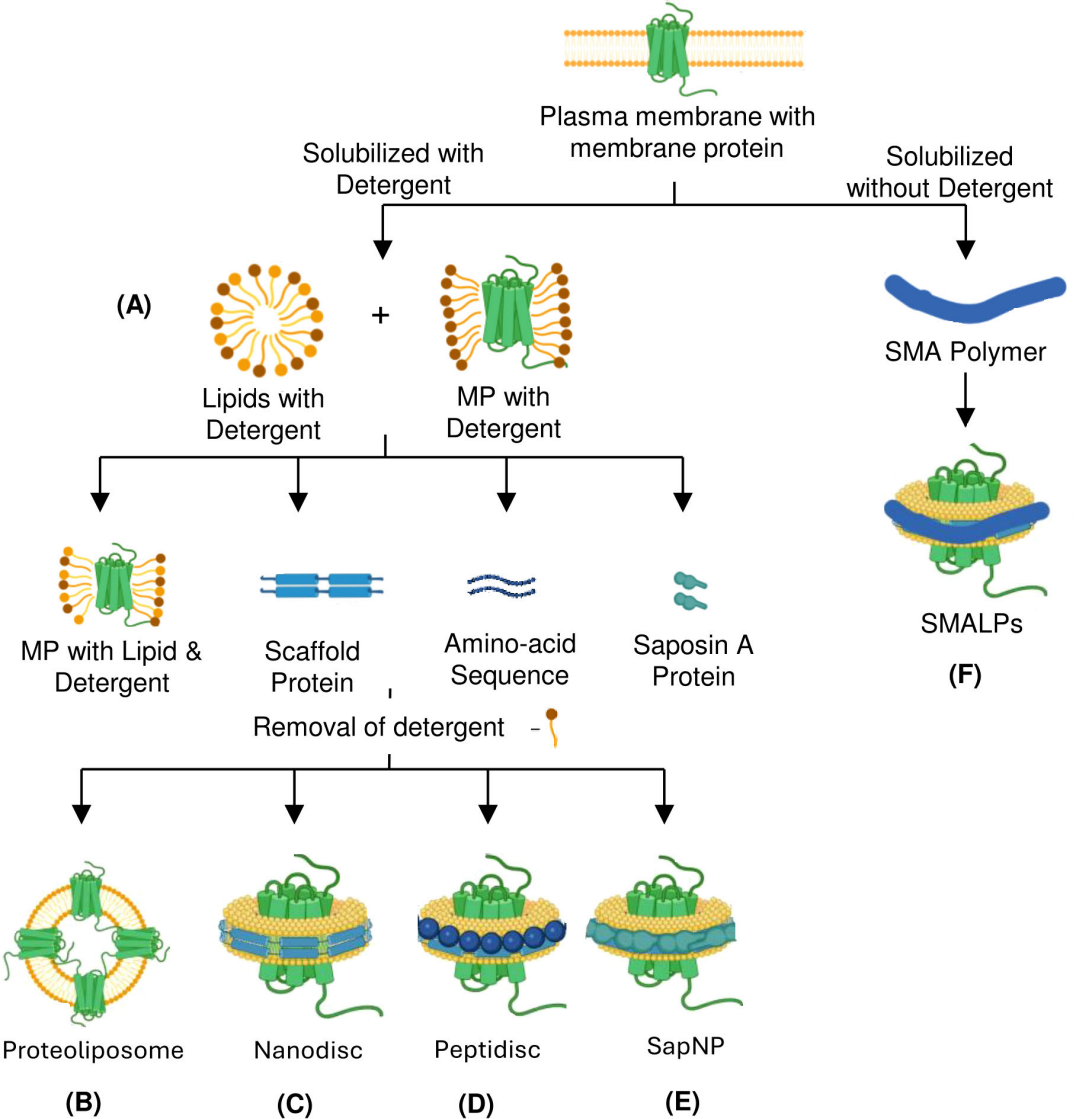
Detergents and nanoformulations-based strategies for preparing membrane protein immunogens using purified proteins. Membrane protein solubilization and purification can be achieved with or without using detergent as an antigen. Mixing detergent-purified protein with lipids into different formats helps in immunization. **(A)** Detergents extract hydrophobic membrane proteins from the plasma membrane by forming micelles. **(B)** Liposomome-based reconstitution of membrane protein. **(C)** Wrapping the lipid bilayer with membrane scaffold protein to form nanodisc. **(D)** Peptidiscs are formed by wrapping with a lipid-bilayer by peptides such as 37-amino acid amphipathic ApoA1-mimetic peptide. **(E)** Saposin A protein is used as a Scaffold Protein to form SapNPs. **(F)** A Detergent-free method that relies on SMA co-polymer form SMALPs. MP, Membrane Protein; SapNP, Saposin lipid Nanoparticle; SMA, Styrene-Maleic Acid; SMALPs, Styrene-Maleic Acid Lipid Particles.

MP solubilization, an essential step for antigen generation, requires masking the hydrophobic surface of integral MPs (29). This can be achieved by micelles which are spherical structures generated by the self-assembly of amphipathic molecules such as surfactants or lipids in aqueous media. They have a hydrophobic core and a hydrophilic outer shell, allowing them to contain hydrophobic molecules within their core. Micelles are highly effective in solubilizing MPs with broad hydrophobic surfaces that are otherwise difficult to retain in solution. Immunization with micelle protein complex, sometimes integrated with adjuvant, elicits an effective immune response (**Figure 2A**). Advances in MP purification utilizing protein-detergent micelles have been fueled by the development of more efficient detergents, such as neopentyl glycols (30). But as the use of detergents has increased in recent years, the obstacles they pose during antibody generation are also being highlighted. The detergents with longer hydrophobic acyl chains are the most stabilizing, but they mask the ECD, which prevents immune response against the masked region. Moreover, after immunization, detergents may dissociate from MP, thus resulting in denaturation and possible conformational epitope loss. Introducing point mutations into the transmembrane helices of proteins is a strategy to overcome the normally low stability of complex MPs in detergent micelles (31). Employing specific point mutations can result in very stable MPs that continue to function in detergent micelles even with short acyl chains. A technique developed by Heptares therapeutics referred to as stabilized receptors or StaR has employed the thermo-stabilized β1-adrenergic receptor in a StaR boost configuration to isolate agonist antibodies and negative allosteric modulators (32).

### 2.2 Proteoliposome

Reconstitution into liposomes is a common method for the functional characterization of MP. The lipid environment is essential for maintaining complex MPs’ correct conformation and function. Liposomes, which mimic natural cell membranes with their lipid bilayer structure, are commonly used as they can encapsulate and transport membrane-associated molecules (33). Typically, liposomes form through self-assembling pure lipids or lipid mixtures (34). MP from various pathogens are being assessed to be incorporated into liposomes as potential vaccines. When compared to detergent-extracted MP, proteoliposomes provide more native-like lipid configuration to complex MPs, enhancing immunogenicity. Liposomes can fuse with the plasma membrane to deliver the antigen directly into cells, allowing it to be processed via the endogenous pathway and thereby eliciting Cytotoxic T Lymphocytes responses (CTL). Overexpressing recombinant MPs in conventional cellular systems can be challenging due to insufficient membrane insertion, precipitation of newly synthesized proteins, or cytotoxic effects caused by significant disruptions to the host cell’s metabolism. Considerable time and effort are required to optimize cell culture conditions, stabilize and solubilize the target MP, purify it, and successfully reconstitute it into proteoliposomes. This system has been used to produce highly effective antibodies targeting the human M2 muscarinic acetylcholine receptor and CCR5 receptor (33, 35).

Another approach to preparing liposomes integrated with MP relies on cell-free translation system. When liposomes are added to a cell-free translation system, the synthesized MP integrates directly into the liposomal lipid membrane, eliminating the need for laborious steps like MP purification and proteoliposome reconstitution (**Figure 2B**) (36). A further modification of the cell-free system incorporates adjuvant-containing liposomes with monophosphoryl lipid A (MPLA), which adjusts the lipid composition to improve the reproducibility and stability of MPs. Additionally, this system produces large amounts of MP antigens, with MPLA acting as a toll-like receptor 4 agonist to enhance antibody production by stimulating B cells (37). However, preparing proteoliposomes for immunization presents significant challenges, including reconstituting detergent-solubilized MPs into liposomes with correct orientation or directly incorporating MPs produced in cells into the liposome structure. Despite these technical difficulties, proteoliposome immunization remains a valuable method for MP immunization.

### 2.3 Nanodiscs

Nanodiscs rely on scaffold proteins and phospholipids to assemble a bilayer that closely resembles the native environment of the MP (28). The nanodisc platform was originally conceptualized as a lipid bilayer stabilized by a hydrophobic belt formed by two copies of amphipathic membrane scaffold proteins (MSPs) (38). MSP self-assembles around the MP in the presence of a lipid, capturing the protein associated with the lipid in the process. SpyCatcher-SpyTag technology is one of the recent advances in MSP engineering, leading to a tenfold higher yield in protein extraction (39). As nanodiscs preserve the structural and functional integrity of the MPs, they provide an interesting approach to developing MP immunogens for antibody generation. In order to prepare a nanodisc-based MP immunogen, the detergent-solubilized MP is incorporated into nanodiscs, which are prepared by assembling lipids such as POPC (1-palmitoyl-2-oleoyl-sn-glycerol-3-phosphocholine) or POPE (1-palmitoyl-2-oleoyl-sn-glycerol-3-phosphoethanolamine) and MSP Apolipoprotein A1 (ApoA1), or its derivatives such as MSP1D1, MSP1E3D1, MSP1D1ΔH5, and MSP2N2 (**Figure 2C**). Following validation, the resulting nanodisc-protein complex is formulated with an adjuvant to maintain stability and enhance the immune response. Nanodiscs incorporated with MPs have been successfully used to generate antibodies against the human apelin receptor (APLNR), immune checkpoint targets like PD-L1, ion channels like K*v*v1.3, (39) and Influenza virus proteins matrix-2 and hemagglutinin, resulting in the increased specificity and affinity of the generated antibodies (38, 40).

### 2.4 Peptidiscs

Peptidisc, an alternative to nanodiscs, developed by Duong et al., is composed of short amphipathic peptides, such as the 37-amino acid ApoA1-mimetic peptide known as the nanodisc scaffold peptide (41). These peptides encircle and embed MPs within a lipid bilayer, mimicking the natural membrane environment (**Figure 2D**) (42). Peptidiscs can reconstitute MPs of varying sizes and topologies from prokaryotes and eukaryotes, offering a distinct advantage over nanodiscs (41). Peptidiscs are particularly advantageous because they provide a more stable and native-like setting for MPs, which are often unstable outside their natural environment. This stability makes them ideal for structural and functional studies, including X-ray crystallography, cryo-electron microscopy (cryo-EM), and drug screening. Despite the potential of nanodiscs for MP stabilization, they face challenges like limited size range, difficulty incorporating large proteins, and complicated preparation. Peptidiscs address these limitations of nanodiscs by using a flexible peptide scaffold that can accommodate even larger proteins with simplified preparation and improved stability.

### 2.5 Saposin lipid nanoparticles

Saposin-based nanoparticles (SapNPs), a new alternative tool for MP stabilization, are a class of nanoparticles derived from saposins (SapA), which are small lipid-binding proteins, forming nanodisc-like structures around MPs. In SapNPs, an MP integrated into a lipid bilayer is enclosed by multiple copies of SapA proteins, which preserves their structural and functional integrity (**Figure 2E**) (43). The flexibility of the SapA scaffold and its ability to adapt to the size of target MPs, accommodating the variable transmembrane regions within the SapNPs, make it an attractive alternative to nanodiscs (43). SapNPs solubilized MPs are used for both immunizations, enabling the immune system to produce specific antibodies against the delivered protein antigens and probes to sort antigen-specific B cells and for *in vitro* high throughput screening experiments (44). SapNPs incorporating malaria antigens have demonstrated the ability to elicit strong antibody responses. These nanoparticles help in better antigen stability and targeted delivery, enhancing immunogenicity against malaria. In cancer vaccine research, SapNPs have been used to deliver tumor-associated antigens. The adjuvanticity of SapA boosts the immune system’s ability to recognize and produce antibodies against these antigens, aiding in tumor rejection (45).

Based on the success of nanoparticle-based membrane solubilization methods, Salipro Biotech has pioneered the development of saposin-based membrane solubilization systems such as Direct MX for the stabilization of MPs in their native lipid environment (46). This method is advantageous because it relies on mild detergent, which preserves weakly associated MPs, helping maintain their native interactions. Like nanodiscs and SMALPs, the Salipro platform has been successfully applied to a wide range of targets, including GPCRs, ion channels, and other integral MPs, facilitating breakthroughs in drug discovery and antibody development.

### 2.6 Styrene-maleic acid lipid particles

SMALPs are similar to nanodiscs as they both utilize lipid-based systems to stabilize MPs in a native-like environment. However, unlike nanodiscs, which utilize MSPs to stabilize lipid bilayer, the bilayer in SMALPs is stabilized by an amphipathic styrene-maleic acid (SMA) copolymer. The copolymer inserts into the membrane and extracts a patch of the bilayer along with the embedded MP, creating a lipid particle around the protein (**Figure 2F**) (47). Unlike nanodisc, SMALPs offer a detergent-free approach to solubilizing MPs and are particularly advantageous for working with larger MPs (48). Similar to nanodiscs, SMALPs have been used to prepare immunogen for antibody production through the preservation of MPs in their natural state. The transmembrane influenza matrix-2 protein, for instance, was integrated into SMALPs, allowing the target domain to be isolated for antibody production. Using this method, a number of antibodies against the cytoplasmic domain of M2 were effectively generated and verified (49). SMALPs have also been utilized in research on human C-type lectin-like receptor 2 (CLEC-2), enabling activation for the production of antibodies (50). This illustrates the adaptability of SMALPs in producing antibodies against MP targets.

## 3 Computer-aided peptide-based immunization

Using peptides for immunization not only alters the need to produce full-length proteins but also directs antibodies to the particular epitopes of interest (51, 52). The peptide-based immunization strategy often depends on synthesizing peptides with short sequences corresponding to the extracellularly exposed portions of MPs or expressing the same regions as fusion proteins or soluble proteins (53). Therefore, peptide-based immunization eliminates the need to purify the entire MP. However, the linear shape of short peptides may lead to discovery of antibodies that do not recognize the native structure of proteins or conformational epitopes unless antibody screening is carried out using native proteins. This problem can be partially resolved by using cyclic or conformationally constrained peptides, which may help retain specific secondary structures by restricting the peptide flexibility, thereby resulting in antibodies recognizing conformational epitopes (54). PEPSCAN recently developed a process known as the chemical linkage of peptides onto scaffolds (CLIPS) that enables the synthesis of mono, di, or tricyclic limited peptides. This technique facilitates the production of conformation-specific antibodies and has been successfully applied to produce functional anti CXCR2 antibodies (55).

Peptide based immunization also allows raising antibodies against post-translational modifications (PTMs) of MPs. PTMs such as phosphorylation, glycosylation, and acetylation can be added to create synthetic peptides that mirror the protein’s native state, increasing the chance of developing high-affinity, functional antibodies against MPs (56). The conventional approach to immunogen designing involves large proteins or whole organisms that lead to an unnecessary antigenic load and induce the chances of allergy (57). This can be avoided by using peptide-based immunogen comprising short immunogenic peptide fragments, capable of evoking strong and targeted immune responses, thereby avoiding the chances of the allergenic response (58). However, the design of peptide-based immunogens for MPs is a challenging task due to the unique structural characteristics of MPs, particularly their hydrophobic regions and complex topologies (59).

**Figure 3:**
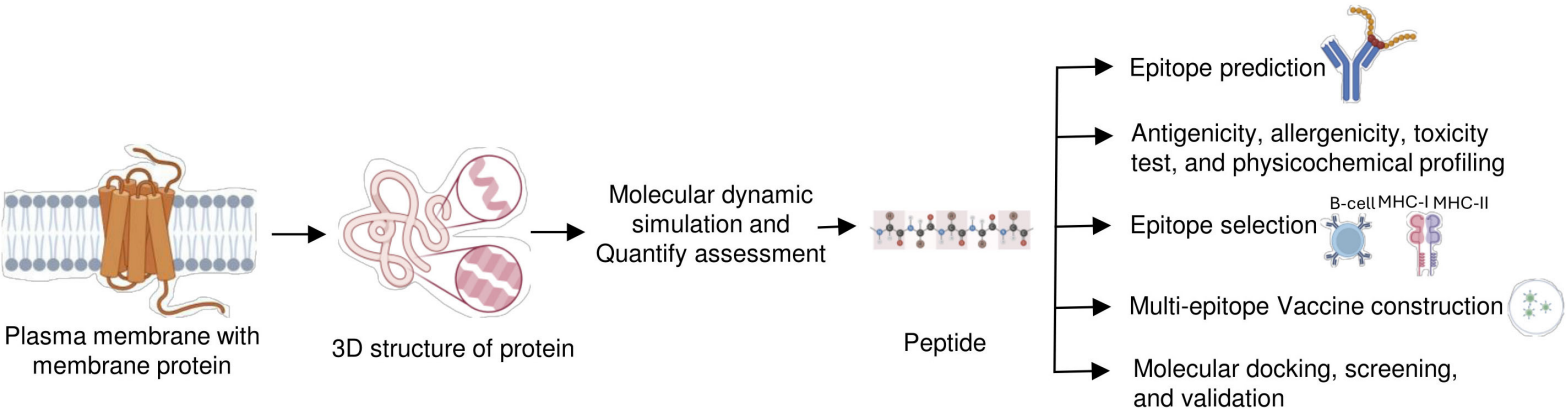
Computer-aided peptide-based immunization strategy. A computational analysis approach is used to develop the peptide-based immunogen that presents ideal features for antibody development. In this approach, first identification and selection of the membrane protein-specific region that will be immunogenic. Immunogenic peptide determination is via antibody interaction and multi-epitope construction, followed by bioinformatic tools and algorithms for testing antigenicity, and physicochemical profiling. Epitopes can then be optimized to best display their antigenic potential.

Computational approaches have emerged as powerful tools to address these challenges, enabling the rational design of immunogens that can evoke a targeted immune response. Computational approaches for epitope prediction can facilitate the selection of both linear and conformational epitopes from trans MPs, paving the way for their development into peptides and soluble proteins suitable for use as antigens in antibody production help in identifying immunogenic regions within MPs (**Figure 3**) (60). Recent advancements in computational immunology, particularly in immuno-informatics and structural bioinformatics, have significantly enhanced our ability to identify promising epitopes on MPs (57, 61, 62).

A critical strategy in the design of peptide-based immunogens is the identification of surface-exposed epitopes, which are required for producing antibodies capable of recognizing and neutralizing native MPs. Tools such as the VaxiJen v2.0 server are used to predict antigenicity by analyzing the physicochemical properties of protein sequences (63). In contrast, AllerTOP v2.0 server is used to distinguish the designed peptide, the immunogenic one, and the non-immunogenic one. These tools enable the screening of a large number of peptide candidates to identify peptides that are more likely to generate immune responses without causing adverse reactions (64, 65).

Traditional methods of epitope identification often rely on protein structures and need to account for the membrane context of proteins. A computational technique such as AlphaFold2 provides a massive transformational advantage over the existing methods (23, 66). AlphaFold2 utilizes the deep-learning algorithm that helps to determine the exact 3D structures of proteins, including the MPs, which are notoriously very challenging to study experimentally. AlphaFold also enhances the precision of predicting surface exposed epitopes in MPs, which is vital for eliciting a robust immune reaction with the accurate model. This capability greatly simplifies the immunogen design process by reducing reliance on experimental techniques and improving epitope prediction precision (67, 68). Although many computational techniques have been created to help in B-cell epitope prediction because experimental methods are expensive and time-consuming. At first, sequence-based techniques were the mainstay of these techniques. But with recent developments in protein structure prediction—like the innovation made by AlphaFold2—structure-based methods have also become strong substitutes to predict B cell epitope. GraphBepi is one such structure-based tool that leverages the advancements in protein structure prediction to improve the accuracy of B-cell epitope identification (69). Additionally, applications such as RoseTTAFold2 and ESMFold also help in modeling the structure of integral MP (70). The peptide-based immunogen formulations are further advanced through innovative methods, such as the design of multi-peptide epitopes derived from various regions of the target protein (71). Additionally, various tools available at the Immune Epitope Database can be employed to predict the immunogenicity scores of single or multi-epitope constructs, enabling the development of immunogens capable of eliciting robust and diverse immune responses (72, 73).

By predicting the most promising epitopes and ensuring they are non-toxic, non-allergenic, and well presented on MHC, computational methods help optimize the design of immunogens that can raise antibodies capable of recognizing native structure of MPs. For example, the peptide-based epitope “YLQPRTFLL” is located within the receptor binding domain (RBD) of SARS-CoV-2 spike glycoprotein, a trans membrane-associated protein crucial for viral entry through its RBD. The computational tools and algorithms predicted that this peptide possesses a high binding affinity to a prevalent HLA a, suggesting its potential to provoke a vigorous CTL (71, 74, 75). In addition, leveraging AlphaFold2 simplifies and accelerates the workflow by effectively forecasting the configuration of MP, which facilitates the logical choice of epitopes that are more likely to provoke robust and targeted immune responses (76, 77). The innovations in the computational design of immunogens and advanced structural prediction technologies such as AlphaFold2 signify a transformative change in developing vaccines and therapeutic antibodies targeting the MPs (66). The ability to efficiently design peptide-reduced risk of adverse immune responses offers promising prospects for the future of immunotherapy and vaccine development.

## 4 Membrane-based immunogen preparations

MPs either partially interact with or are fully embedded within the cellular membrane, characterized by their hydrophobic transmembrane domains, which cause them to aggregate, particularly when misfolded (78). Therefore, micelles, cell membranes mimicking lipid bilayer systems, or polymers are often necessary to solubilize and stabilize MPs. These methods enable more precise immune responses by maintaining the function and stability of MPs. The key membrane-based immunization strategies to produce anti-membrane antibodies are described below.

### 4.1 Whole-cell immunization

**Figure 4:**
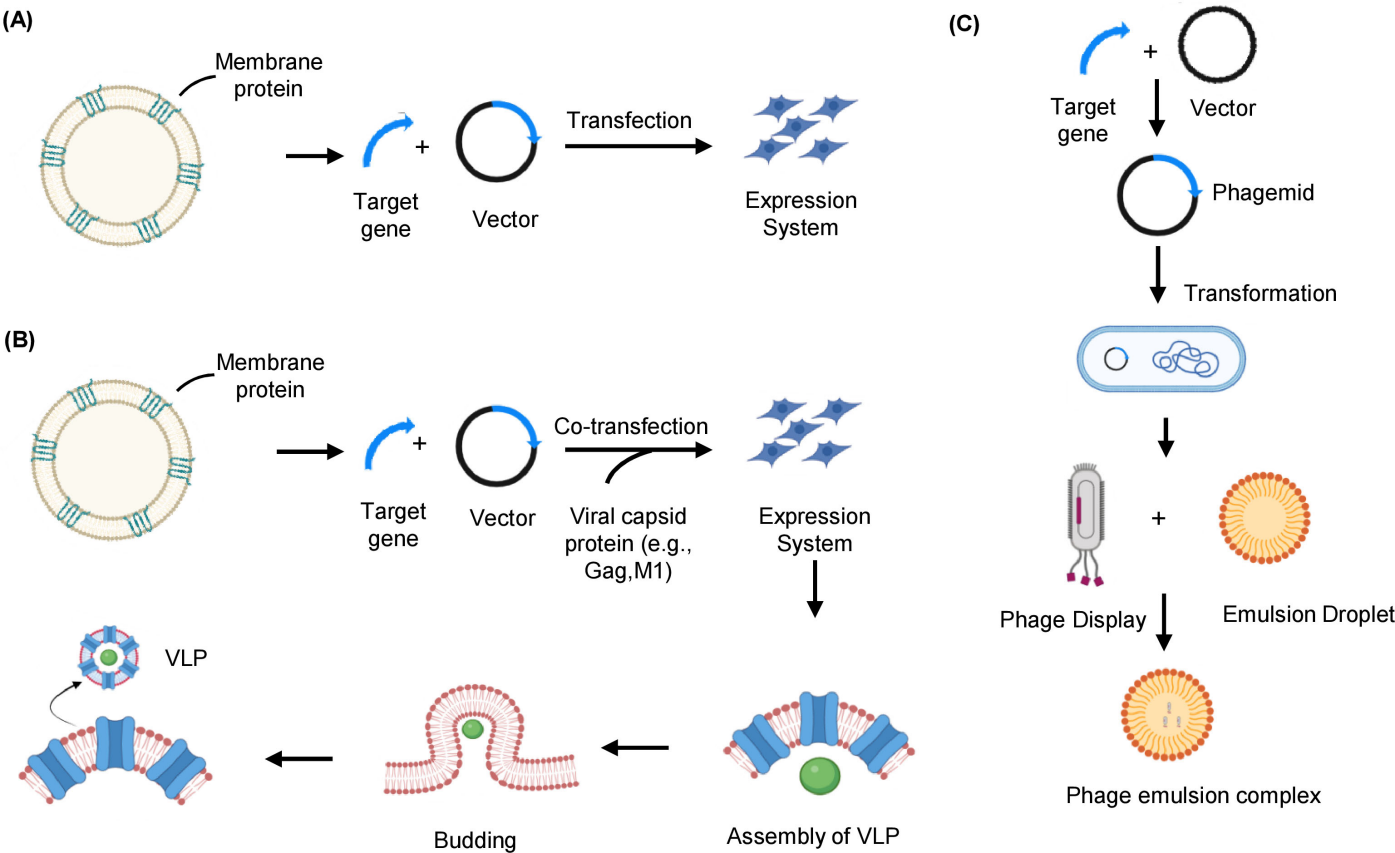
Native membrane-based strategies to express and prepare membrane protein immunogens. **(A)** Whole cells overexpressing the target membrane protein of interest can be used as immunogens. The whole cell’s entire proteome is injected into the host cell’s body, which considers it non-self and develops an immune response. **(B)** VLPs are produced by co-expressing the target antigen with viral capsid protein in the mammalian expression system. The capsid protein self-assembles at the plasma membrane, where the membrane protein of interest is overexpressed and buds off to form a VLP that serves as an immunogen for delivery into a host cell. **(C)** The target membrane protein gene segment is integrated with the phage genome, expressed on the phage coat. The phage displayed with the target membrane protein is encapsulated in an emulsion droplet to immunize the host. VLP, Virus-like particle.

Whole-cell immunization effectively generates antibodies against MPs by expressing them in their native conformation on the cell surface to overcome the challenges of *in vitro* antigen preparation, making them suitable for a wide range of applications. Preparing pure, homogeneous, and conformationally stable MP antigens from plasma membranes is challenging. Hence, whole-cell antigen presentation preserves the MP in a native environment with correct and functional folding. In this approach, cells are engineered to express the MP of interest and are subsequently used to immunize animals (**Figure 4A**). Whole-cell immunization targets relevant functional epitopes of MPs that play essential physiological roles, helping the immune system to produce accurate antibodies. A key challenge with whole-cell immunization is the limited expression of MPs on the cell surface, which often necessitates strategies for their overexpression. Recent developments of flow cytometry-based transfection strategies such as MaxCyte flow electroporation efficiently express and isolate cell expression target proteins that are otherwise difficult to express (79). Such a strategy can achieve a transfection efficiency of up to 2 × 10^11^ cells without significant loss of viability to create stable cell lines (79). Such advanced strategies can successfully transfect and isolate cells even when the transfection efficiency is very low (80). Since most MP do not exist in abundance, overexpressing relevant epitopes in an expression system can increase the chances of immunogenicity.

Whole-cell immunization has successfully generated antibodies against various MPs, such as the HER2 protein in breast cancer and GPCRs. A monoclonal antibody 4D5, targeting the ECD of the HER2 protein, effectively inhibits the growth of HER2 overexpressing breast tumor cells and enhances their sensitivity to TNF-α (18). Immunizing with cells that express membrane peptides or proteins has successfully generated antibodies that modulate the function of ion channels, or GPCRs (51). For example, researchers generated antibodies against GPCR 5 by co-transfecting cells with the receptor peptide and receptor activity-modifying proteins, enabling the expression of the MP region. In contrast, antibodies targeting ion channels like Orai-1 and P2X7 have been developed for treating autoimmune and inflammatory diseases, respectively (81, 82). Bacterial ghost platforms, consisting of intact bacterial cell envelopes emptied of their contents, are also utilized to express or deliver various antigens, plasmid DNA encoding protein epitopes or present MP for immunization. The ghost vaccine method serves as an efficient carrier platform, addressing the potential poor immunogenicity of protein subunits and DNA-encoded antigens by effectively delivering them to antigen-presenting cells (83). Noncapsular bacterial endotoxin surrounding some bacteria can also be used to elicit an immune response. For example, to combat *Klebsiella pneumoniae* infections, a natural conjugate was developed by combining the bacterium’s O-antigen, a surface component that triggers an immune response, with bacterial outer MPs to strengthen the immune response (84). Although whole-cell immunization approach holds great potential, developing stable cell lines that express MPs at sufficient levels requires significant time and effort. However, once established, a single cell can be clonally expanded to consistently produce high MP levels, which researchers can use for antibody production and store indefinitely (85). Human Embryonic Kidney 293 (HEK-293) cells (86) and insect cells are commonly used to generate transient and stable expression systems to overexpress MPs. Whole-cell immunization using an engineered cell line is helpful for MPs that are challenging to isolate or purify, as it allows MPs to remain in their natural conformation on the cell surface (45).

### 4.2 Virus-like particle

In addition to other lipid bilayer-based membranous formats, besides proteoliposomes and nanodiscs, which resemble native biological membranes, including virus-like particles (VLPs) (87). As MPs are typically present in low quantities in their natural hosts, it is necessary to overexpress them in heterologous systems for sufficient production. VLPs are hollow structures consisting of a viral core protein encapsulated by portions of the cell membrane with MPs and receptors expressed in their native conformation. VLPs mimic the native structure of viruses but are non-infectious due to the absence of viral genetic material (88). These vesicular structures, typically 20 to 200 nm in diameter, are enriched with the MP of interest up to 100-fold and can be generated from a cell population that overexpresses the protein on its surface (89). The VLPs are produced by transfecting insect or mammalian cells with the retroviral core protein (gag) expression vectors. The capsid protein produced from the transfected vector spontaneously self-assembles to form VLPs. As gag protein naturally assembles at the plasma membrane and buds off from host cells that overproduce the target MP, VLPs displaying MP of interest (MP-VLPs) can be produced by co-expression of gag protein with the MP of interest (**Figure 4B**) (87, 90). A key step in successful VLP production is selecting the appropriate expression host system. The choice of an ideal system depends on various factors, such as the specific target, required quantity, and intended downstream applications.

There are several benefits to using VLPs for phage display (91). Compared to other carriers, VLPs offer a higher density of foreign proteins per particle and maintain a unique 3D structure, which is crucial for presenting conformational epitopes. As compared to whole cells, VLPs can provide a high concentration of target MP with fewer host cell MPs. VLPs can also be stored for prolonged periods at -80°C without significant degradation or loss of functionality when compared with other membrane-based formats (92). Due to the viral gag protein functioning both as an adjuvant and in VLP formation, the use of external adjuvants or toxins to enhance immunogenicity is not required. Preserving MPs in their natural phospholipid bilayer in a conformationally stable state is a significant advantage of this strategy, making VLPs suitable for both immunizations as well as antibody screening and validation (93). VLPs have been successfully used for both vaccination and antibody production for diagnostic purposes. For example, neuraminidase containing N1 VLP derived from the H1N1 influenza virus, when immunized in mice, induced virus-specific antibodies as well as reduced NA inhibition activity (94). The E protein of the Zika virus (specifically domain ED-II), the E2 protein of the chikungunya virus, and the L1 or L2 proteins of the human papillomavirus are utilized as antigens in VLP vaccines (95, 96).

### 4.3 Phage emulsion technique

The use of bacteriophages, viruses that infect bacteria, as direct immunogens are very well understood, but their use as a tool to generate antibodies indirectly acting as a carrier is also crucial. The phage display technique is based on genetically altering the phage DNA to allow the expression of a segment of peptide or protein on the phage surface (97). The DNA sequence of the protein of interest is inserted near the nucleotide sequence, which encodes for one of the phage coat proteins. Following phage infection, the inserted peptide or protein is displayed on the phage surface as a combined product of the genes generating the coat protein and the cloned protein (**Figure 4C**) (98).

The phage emulsion technique is a well-adapted version of the phage display technique for antibody generation. This method involves integrating a protein, peptide, or even synthetic epitope on the surface of bacteriophages and mixing them with an aqueous-organic phase system to form a stable emulsion. The bacteriophage emulsion complex is further used for immunization. The bacteriophage particles are effective carriers that closely resemble natural infections by displaying the associated antigens in a 3D conformation (99). The immune system perceives the phages as foreign particles and immediately starts an, immune response against the antigen they display. The emulsification procedure is crucial to create a stable environment for antigen and bacteriophage association. Thus, the antigen’s exposure to the immune system is prolonged, leading to a robust immune response and antibody generation (100). This method offers a strong foundation for creating new immunogens and vaccines and studying the relationships between specific antigens and the immune system (101). Phage micro-emulsion technology provides many essential benefits over existing phage-display techniques. One benefit is that each clone’s signal is enhanced because all phage copies are contained in a single droplet (102). However, there are still many challenges to the phage emulsion technique, such as difficulty in emulsion formation utilizing complex MPs as targets, high cost, etc. (103), which future research is expected to address. The filamentous phages are generally employed in the phage emulsion technique, but recently, various other bacteriophages are being modified, such as lytic phages, which can display more prominent antigens with their ability to carry larger foreign DNA fragments (104).

### 4.4 Exosomes

Exosomes are extracellular vesicles that carry a variety of cellular components, including DNA, RNA, MP, lipids, and both cytosolic and cell-surface proteins. Membranous nanovesicles, typically 50-100 nm in diameter, form within late endosomal compartments through the invagination of multivesicular bodies (MVB) membranes. The endosomal membranes involved in their formation are known as intraluminal vesicles (ILVs). Mature MVBs undergo exocytosis to release ILVs as exosomes into the extracellular environment after fusing with the plasma membrane (105, 106). As a result of this formation process, exosomes have a membrane orientation similar to the native plasma membrane, with ECDs facing outward and cytosolic proteins, along with small RNAs, enclosed within the lumen. After their secretion, exosomes protect their cargo and deliver it to the recipient cells by endocytosis or fusion without compromising the intrinsic function of the cargo (107). These exosomes involve cell-to-cell communication, immune response modulation, and intercellular signaling and are of high pathophysiological relevance in various diseases, including cancer (108, 109).

Exosomes offer several advantages as immunogens in anti-MP antibody production, including the lack of need for sequence modification, preservation of the native membrane environment, high intrinsic immunogenicity that elicits strong immune responses, and excellent stability for both short and long term storage. Additionally, exosomes do not require adjuvants and exhibit minimal cytotoxicity. Intracellular exosomes can be isolated and used to deliver proteins and RNA from other cells into ex vivo cell cultures. Exosomes can be purified from *in vitro* cultures of various cell types and can be loaded with a diverse range of biological molecules, including cytosolic proteins, membrane receptors, and nucleic acids (110).

MP bearing exosomes have successfully contributed to the development of numerous anti-GPCR antibodies (111, 112). Notably, exosomes secreted from dendritic cells, known as dexosomes, demonstrate exceptional immune capabilities *in vivo* (113). An extracellular vesicle-based technology enables the selective recruitment of viral membrane antigens containing WW domains onto the surface of WW domain–activated extracellular vesicles (WAEVs). By fusing viral MPs or peptides to WW domains, MP antigens can be efficiently displayed on the surface of WAEVs, eliciting robust antibody production that explicitly targets the corresponding viruses. This approach likely preserves the natural conformation of MPs, as specific antibodies, including neutralizing antibodies, recognize them (114). A newer approach in membrane engineering involves hybrid exosomes formed by merging exosome membranes with liposomes through the freeze-thaw method (115). The exosome liposome fusion technology is promising for loading therapeutic compounds into exosomes, making it easier for MPs to be incorporated into the exosomes. However, limitations of using exosomes for anti-MP antibody production include limited yield, purity issues, heterogeneity, complex production processes, and potential regulatory challenges.

### 4.5 Genetic immunization

**Figure 5:**
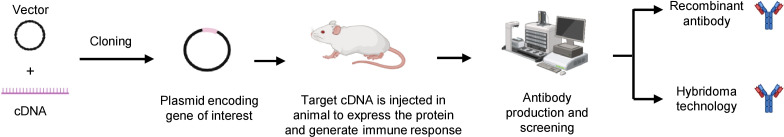
DNA-based immunization to generate membrane protein antibodies. DNA encoding membrane protein can be introduced directly into a host, allowing it to express the intact immunogens and generate antibodies against it.

The genetic immunization approach, involving DNA and mRNA immunization, relies on expression vectors or utilizes synthetic mRNA sequences to produce encoding the target immunogen peptides or proteins directly within the host. These expression vectors or mRNA are typically delivered to the host intradermally through various methods such as injection of naked DNA or mRNA using a simple needle or biolistic delivery of DNA-coated particles into dermal cells, transdermal patches, and electroporation-based and viral-based methods (116–118). Each of these studies used DNA of mRNA sequences encoding for soluble proteins or the soluble domains of membrane-anchored proteins (**Figure 5**). Genetic immunization overcomes the problem associated with peptide or protein-based immunization for antibody production against MPs because the gene-immunized host naturally produces, folds, and alters the MP antigen in its natural membrane context.

One of the challenges of traditional protein-based immunization is the production of full-length MP immunogens in their native, membrane-associated form using recombinant protein techniques. The problem is even more significant, particularly for proteins like multispanning transmembrane proteins such as GPCRs and ion channels. DNA immunization overcomes these obstacles by enabling *in vivo* expression of full-length proteins, eliciting a targeted immune response against the MP by directly delivering DNA to cells, and inducing the formation of the desired proteins, effectively turning the cells into a source of antigen production to stimulate an immune response (118). DNA-based immunization for SARS-CoV-2 envelope and MPs in mice, provided protection against the disease (119). DNA expression vectors coated with gold particles injected into shaved and depilated skin cells of camelid using gene gun delivery methods expressed MP on transfected cells and led to the generation of nanobodies targeting MPs (120). RNA immunization reduces the risk of DNA integration into the host genome by directly delivering the target protein to the cytosol, improving the safety profile of nucleic acid, and enabling quicker immune response. Recently, a self-amplifying RNA-based immunogen containing the additional sequences of nsP1-4 proteins allowed their self-replication within the host cells. This helps in achieving a robust immune response with smaller doses compared to conventional RNA vaccines (121). Taken together, DNA and RNA-based immunization strategies, have demonstrated significant potential in inducing immune responses against MP.

## 5 Conclusion

Producing functional anti-MP antibodies requires the preparation and immunization of the protein of interest in its native form. In this review, we discussed various strategies for preparing and immunizing MP so that antibodies produced can recognize both linear as well as conformational epitopes of MPs, for therapeutic purposes (**Table 1**). The choice of antigen format for antibody discovery is dependent on compatible solubilization and purification methods for the target of choice. Selecting an appropriate method for immunogen production is challenging, as each approach comes with distinct advantages and limitations and the downstream antibody discovery platform used. The formats used range from simple soluble regions of the protein, such as soluble ECDs or peptides, to full-length MPs purified in detergent or lipidic environments, to complex membranous environments, such as VLPs, exosomes, and whole cells. Although generating specific antibodies from peptide-based immunizations eliminates the need to produce full-length transmembrane protein, the linear structure of peptides restricts the establishment of conformational epitopes and makes it more difficult to find effective antibodies. These problems can be partially tackled by complementing peptide-based immunogen design with computational modeling and prediction tools. A more recent advancement in AI-based structure prediction tools, such as AlphaFold2, RoseTTAFold2, and ESMFold, has great potential to predict the 3D structure of proteins directly from the amino acid sequence for more precise identification of immunogenic areas that can be used as antigens. Further advancements in AI-based tools make *de novo* protein design, epitope, and paratope identification, soluble analogous of MP, and native conformation analysis a realistic vision.

**Table 1:** Comparison for different approaches for MP immunogen.

Method	Mechanism	Advantages	Limitations	Antibodies against targeted antigen	References
Detergent Micelles	Solubilization of hydrophobic MPs through amphiphilic molecules	Most widely used and easy to apply technique to solubilize MP	Masking of extracellular loops disrupts the binding of antibodies.	Nb80 antibody (Beta 2 adrenergic receptor)	(122)
Proteoliposome	Purified MP encapsulated in the liposome	Liposomes mimic natural cell membrane-associated molecules	Reconstituting detergent-solubilized MPs or cells produced into liposomes.	1D4 monoclonal antibody (CCR5 protein), Recombinant antibody (Human M2 muscarinic acetylcholine receptor)	(33, 35)
Nanodiscs	Assembly of MP with Scaffold proteins and phospholipids resembling bilayer	Increases specificity and affinity of the generated antibody	Unable to mimic membrane curvature and lipid symmetry in the cellular membrane.	Single-domain antibody (Human apelin receptor)	(38, 40, 123)
SapNPs	Saposin A(SapA) forms a nanodisc-like structure along with MPs.	Preserve weakly associated MPs	Difficult to obtain in biologically active format for antibody discovery experiments.	Antibodies against malaria antigen, antibodies against HIV-1 spike protein	(45, 124)
SMALPs	Styrene-maleic acid (SMA) copolymer stabilized with MPs.	Only detergent-free approach to solubilizing MPs.	Solubilization efficiency is lower than commonly used detergents.	Anti-CLEC-2 AYP2(C-type lectin-like receptor 2)	(48, 50)
Computer-aided peptide-based immunization	Computational tools and algorithms are used to design the peptide-based immunogen.	Cost-effective and less time-consuming than other methods.	Incomplete database in these tools may lead to selection of non-immunogenic or non-functional epitopes	CR3022 monoclonal antibody (Spike protein SARS CoV-2short-chain amino acid residues YLQPRTFLL of RBD region)	(125–127)
Whole-cell immunization	Overexpress the target MP antigen in whole-cells using expression plasmid.	Target antigen presentation in its native form.	Developing stable cell lines, Cell proteins will compete with overexpressed target antigens for antibody binding.	Monoclonal antibody 4D5 (HER 2 protein), Orai-1 antibodies, Anti-ETBR antibodies (Endothelin A receptor)	(18, 81)
VLP	Overexpress the target MP antigen in a hollow structure consisting of a viral core protein to mimic lipid bilayer.	Offer a higher density of foreign proteins per particle and maintain a unique 3D structure. More stable and viable than micelles and liposomes.	Cost inefficient, optimizing each receptor target for high expression can be a labor-intensive process.	Monoclonal antibodies (Glucose transporter, E protein of the Zika virus, E2 protein of the chikungunya virus. L1 or L2 proteins of the human papilloma virus)	(95, 96, 128)
Exosomes	Exosomes have a membrane orientation similar to the native plasma membrane.	Preservation of the native membrane environment, high intrinsic immunogenicity that elicits potent immune responses.	Limited yield, purity issues, heterogeneity, and complex production processes.	Monoclonal antibodies (Prostaglandin F2 Receptor Negative Regulator, brain abundant membrane attached signal protein-1Lysosome-associated membrane protein-2b)	(129, 130)
Genetic immunization	Utilizes synthetic DNA or mRNA sequences to produce target immunogen peptides or proteins directly within the host.	*In vivo* expression of full-length proteins. Targeted immune response by directly delivering DNA to cells.	Require a large dose to be effective.	DNA vaccines (rAHA_2144, rpilQ),13E11 monoclonal antibody (CCX-CKR), Monoclonal anti-claudin 1(protein claudin-1)	(131, 132)

Biophysical techniques like X-ray crystallography, single-particle cryo-electron microscopy, and receptor-ligand binding assays are used to determine 3D structures. However, these techniques do not work in the native environment; therefore, the protein must be separated from the membrane and examined *in vitro* in a lipid or detergent environment. MP extracted through traditional methods such as detergents may destabilize MP as well as mask key extracellular loops of MPs and their dissociation from the membrane. Novel technologies like amphiols, nanodiscs, and SMALPS help overcome these limitations by replicating the composition of plasma membranes. While nanoparticle-based technologies have been widely used in MP structure elucidation (133), their role in the immunization of MPs remains limited. Despite their success in structural studies, the limited use of nanoparticle-based technologies for immunization is largely due to stability issues post-immunization. Nanoparticle-based formulations may also sometimes induce aggregation or alter the native conformation of MPs, affecting both immunogenicity and specificity. Additionally, maintaining the structural and functional characteristics of MPs throughout the MP reconstitution process is still very challenging. The variability incorporated during immunogen preparation can affect reproducibility in immunogen design. In order to minimize conformational changes, aggregation, and unpredictability, it is imperative to develop better membrane-mimetic systems that more precisely mimic the native lipid environment of MPs. Cryo-electron microscopy and other advanced characterization techniques are crucial for evaluating the structural consistency and integrity of MPs in different formulations. The development of innovative adjuvants tailored for MP immunization, as well as scalable and reproducible production techniques, will help ensure reliable antibody production against MPs.

Overexpressed MPs can be utilized for immunization either as whole cells expressing MPs or as MP-containing VLPs and exosomes produced by cells. Although VLPs evoke powerful immune responses and are more robust than whole-cell immunization, allowing fewer non-relevant targets on the surface, they can be expensive and time-consuming to produce. While exosomes can deliver MP in native form with a topology similar to the native plasma membrane, they face challenges such as heterogeneity, complex isolation processes, and limited scalability. Cell-free expression systems has emerged as powerful techniques for the expression of MP. Recent developments in detergent-free modified cellular lysates enable cell-free expression of target proteins, which can be coupled with direct reconstitution of newly synthesized proteins into membrane vesicles such as liposomes. This approach avoids common challenges such as poor membrane insertion, precipitation of newly synthesized proteins, or cytotoxic effects caused by excessive strain on the host cell’s metabolism. The chosen, optimized antigen formats can then be employed to drive antibody discovery through the appropriate use of strategies that leverage display technologies, B cell platforms, hybridoma, or a combination of these approaches to guarantee the isolation of a diverse panel of antibodies.

The effort required to create purified proteins or native membrane structures to generate antibodies can be bypassed by genetically immunizing the host organism. The SARS-CoV-2 pandemic demonstrated the effectiveness of RNA-based vaccines developed in a significantly shorter timeframe. However, challenges remain in ensuring that the expressed protein is properly processed, presented, and elicits a strong immune response. Future improvements in RNA delivery systems, such as more efficient lipid nanoparticles, and improvements in RNA synthesis and delivery technologies, are needed to make this approach for broader implementation. Further, the development of a well-optimized standard protocol to select the optimal antigen format and antibody generation platform for specific targets is also necessary. In addition, developing better strategies for screening membrane antibodies is another critical area in enhancing the production of specific antibodies. These advancements will pave the way for more efficient, accurate, and scalable antibody-generation processes, particularly for challenging targets like MPs.
